# Association Between Non-Cystic Fibrosis Bronchiectasis and Quality of Life: A Single-Center Cross-Sectional Study

**DOI:** 10.7759/cureus.14231

**Published:** 2021-03-31

**Authors:** Sheetal Chaurasia, Alamelu Haran, Anish Reddy, Pavny Chawla

**Affiliations:** 1 Pulmonary Medicine, Manipal Hospitals Whitefield, Bangalore, IND; 2 Pulmonary Medicine, Vydehi Institute of Medical Sciences and Research Center, Bangalore, IND; 3 Respiratory Diseases and Sleep Disorders, Artemis Hospital, Gurgaon, IND

**Keywords:** bronchiectasis, quality of life, exercise capacity

## Abstract

Introduction

Bronchiectasis is a chronic respiratory disease that can affect patients of all ages and significantly impact the quality of life (QOL) in patients who suffer from it. In spite of its widespread prevalence, and the significant impact on QOL, data on the quantitative impact of bronchiectasis on QOL is lacking. The Quality of Life-Bronchiectasis (QOL-B) is a self-administered patient-reported outcome measure, that was recently developed as a response to the emergent need for such measurement tools to study the impact of bronchiectasis on QOL.

Methods

We conducted a single-center cross-sectional study to study the correlation between QOL and various other outcome parameters such as exercise capacity, lung functions, co-morbidities, inflammatory markers, and body mass index (BMI). The secondary outcome was to find out various determinants of quality of life in non-cystic fibrosis bronchiectasis (NCFB).

Results

Forty-four patients who determined the pre-determined criteria for NCFB were enrolled in this study. This study demonstrated a significant impact on the QOL of NCFB patients based on the QOL-B scoring system. Almost all domains of QOL-B were found to be adversely impacted as measured by one or more of the outcome parameters but the FEV1, age, colonization, extension, dyspnea (FACED) score, bronchiectasis severity index (BSI) score, six-minute walk test (6MWD), and FEV1 showed associations across most scales while the other outcome parameters showed varying associations.

Conclusions

The QOL is significantly reduced in NCFB and it may be quantified using the QOL-B questionnaire. The impact on QOL in NCFB may be assessed using validated tools such as the FACED and BSI scoring systems, as well as other well-established outcome parameters like 6MWD and FEV1 predicted.

## Introduction

Bronchiectasis is a chronic respiratory disease that can affect patients of all ages. It is more prevalent in women as the age advances, and there is an increasing trend in the prevalence recently [1]. The underlying etiology in bronchiectasis could involve cystic fibrosis (CF), alpha-1-antitrypsin deficiency, primary ciliary dyskinesia, allergic bronchopulmonary aspergillosis, connective tissue disorders, inflammatory bowel diseases, congenital malformations, aspiration, humoral immunodeficiency, post-infectious, and finally idiopathic cases [2]. The pathophysiology behind bronchiectasis includes chronic inflammatory micro-environments that can trigger the breakdown of the airway tissue. The mechanism remains more or less the same in both CF and non-CF bronchiectasis, with a complex interplay between infection and inflammation that feeds a pro-inflammatory vicious cycle. This cycle progressively drives the formation of bronchiectasis and subsequent destruction of the pulmonary architecture [3].

Patients generally present with daily excessive sputum and other associated symptoms, recurrent chest infections and as a consequence of the chronic course with recurrent exacerbations, am impaired health-related quality of life (QOL) [2]. In spite of its widespread prevalence, and the significant impact on QOL, data on the quantitative impact of bronchiectasis on QOL is lacking. The Quality of Life-Bronchiectasis (QOL-B) is a self-administered patient-reported outcome measure, that was recently developed as a response to the emergent need for such measurement tools to study the impact of bronchiectasis on QOL. It is useful in the assessment of the symptoms, functioning, and health-related quality of life for non-CF bronchiectasis patients. It includes 37 parameters on eight scales including respiratory symptoms, physical, role, emotional, and social functioning, vitality, health perceptions, and treatment burden [4].

In this study, we looked at the various determinants of QOL in non-cystic fibrosis bronchiectasis (NCFB) and the correlation of QOL with clinical, radiological, and physiological parameters in bronchiectasis.

## Materials and methods

This study was conducted as a single-center cross-sectional study in a 1000 bedded tertiary care hospital in the state of Karnataka, India between January 2016 and December 2016. Forty-four consecutive adult patients above the age of 18 years, who had NCFB and were clinically stable were included in this study after informed consent. The exclusion criteria consisted of other co-existent respiratory disorders or chronic debilitating disorders involving any system which could confound results, pregnant women and children, and patients who were not willing to participate in the study. 

The primary objective of this study was to study the correlation between QOL and various other outcome parameters like exercise capacity, lung functions, co-morbidities, inflammatory markers, and BMI. The secondary outcome was to find out various determinants of quality of life in NCFB.

The diagnosis of NCFB was confirmed by high-resolution computerized tomography (HRCT) thorax. After a detailed history and relevant investigations, the following outcome parameters were obtained: Modified Medical Research Council (MMRC) grading, body mass index (BMI), six-minute walking distance (6MWD), C-reactive protein (CRP), and the forced expiratory volume in one second (FEV1). The severity of NCFB was stratified using two validated scores: the FEV1, age, colonization, extension, dyspnea (FACED) score and the bronchiectasis severity index (BSI) [2]. The FACED score is used to predict the risk of five-year mortality. It is calculated using FEV1% predicted, age, chronic Pseudomonas colonization, the extent of bronchiectasis, and the Medical Research Council Dyspnea Scale score [2]. The BSI was calculated using an online tool. The determinants of BSI include age, body mass index, FEV1% predicted, Medical Research Council Dyspnea Scale score, lobes affected, and evidence of chronic bacterial infection [2]. 

The QOL was assessed using the QOL-B questionnaire (Version 3.0). The QOL-B questionnaire is a self-reported questionnaire consisting of 37 items based on eight scales (respiratory symptoms {RS}, physical {P}, role {R}, emotional {E}, social functioning {S}, vitality {V}, health perceptions {HP}, and treatment burden {T}) [4]. Data were collected on the number of exacerbations, the use of antibiotics, oral steroids, hospitalizations, or emergency department admissions in the previous month. The outcome parameters were correlated with each scale of QOL- B separately. 

Statistical analysis was done by STATA Version 11.2 (StataCorp, College Station, TX). Descriptive statistics were used to compute baseline data according to data distribution. Mean differences were compared using t-test or analysis of variance (ANOVA) as applicable and median using Kruskal Wallis test. Correlation between different parameters was done by Pearson or Spearman correlation coefficient depending on the distribution of the values. Predictors of different domains of quality of life were found out by multivariate regression analysis.

Even though it was a questionnaire-based study, an ethical committee clearance was obtained for this study, and all patients were enrolled after informed consent. The study is reported in accordance with the Strengthening the Reporting of Observational Studies in Epidemiology (STROBE) statement [5].

## Results

Forty-four patients who determined the pre-determined criteria for NCFB were enrolled in this study. There were 24 (55%) males and 20 (45%) females with a mean age of 43.4 ± 11.7 years. There were four patients (9%) in the MMRC grade zero category, eight patients (18%) in the MMRC grade one category, 13 patients (30%) in the MMRC grade two category, 15 patients (34%) in the MMRC grade three category, and four patients (9%) in the MMRC grade four category. Comparison between QOL-B scales and MMRC grades showed significant associations between higher MMRC grades and poorer scores in respiratory symptoms, physical, role, emotional, social functioning, vitality, health perceptions, and treatment burden scales (Table 1). 

**Table 1:** Quality of life compared with MMRC MMRC: Modified Medical Research Council

	MMRC grade					
	0	1	2	3	4	p-Value
	Mean ± SD	Mean ± SD	Mean ± SD	Mean ± SD	Mean ± SD	
Physical	53.75 ± 20.20	67.45 ± 21.46	49.72 ± 20.96	30.11 ± 23.09	6.67 ± 0	<0.001
Role	60.01 ± 19.60	62.46 ± 15.48	54.36 ± 29.29	43.55 ± 18.49	33.33 ± 9.43	0.125
Vitality	63.84 ± 16.63	53.88 ± 23.05	54.33 ± 20.06	36.29 ± 19.03	22.83 ± 16.29	0.008
Emotional	70.84 ± 22.05	77.0 ± 16.44	66.61 ± 19.17	57.22 ± 23.33	31.25 ± 7.98	0.008
Social	66.68 ± 29.66	64.58 ± 19.79	64.74 ± 27.24	54.44 ± 19.89	30.83 ± 17.92	0.106
Treatment burden	32.22 ± 49.32	75.95 ± 20.45	64.15 ± 27.14	62.18 ± 20.11	69.42 ± 10.53	0.172
Respiratory symptoms	91.42 ± 7.68	70.67 ± 19.22	60.49 ± 17.68	49.24 ± 20.83	23.17 ± 18.77	<0.001
Health perceptions	68.75 ± 24.88	52.21 ± 20.08	51.66 ± 20.68	44.53 ± 21.74	39.50 ± 14.43	0.234

Sixteen patients (36%) had a mild FACED score, eight patients (18%) had a moderate FACED score, and 20 patients (45%) had a severe FACED score. Comparison between QOL-B scales and FACED scores showed significant associations between higher FACED score grades and poorer scores in respiratory symptoms, physical, role, emotional, social functioning, vitality, health perceptions, and treatment burden scales (Table 2). Twelve patients (28%) had a low BSI score, nine patients (20%) had an intermediate BSI score, and 23 patients (52%) had a high BSI score. Comparison between QOL-B scales and BSI scores showed significant associations between higher BSI score grades and poorer scores in respiratory symptoms, physical, role, emotional, social functioning, vitality, health perceptions, and treatment burden scales (Table 3).

**Table 2:** Quality of life compared with a grade of FACED score FACED: FEV1, age, colonization, extension, dyspnea

	FACED score			
	Mild	Moderate	Severe	p-Value
	Mean ± SD	Mean ± SD	Mean ± SD	
Physical	58.0 ± 21.09	59.13 ± 20.53	23.92 ± 20.78	<0.001
Role	61.25 ± 25.08	59.96 ± 19.81	38.67 ± 14.92	0.003
Vitality	59.25 ± 17.17	52.79 ± 23.57	32.89 ± 18.59	0.001
Emotional	73.92 ± 20.12	65.54 ± 20.98	52.08 ± 21.27	0.011
Social	66.15 ± 27.80	70.83 ± 13.36	46.99 ± 19.91	0.013
Treatment burden	58.69 ± 34.80	66.67 ± 19.98	65.38 ± 18.66	0.728
Respiratory symptoms	72.39 ± 17.99	65.87 ± 15.91	42.45 ± 22.84	<0.001
Health perceptions	56.56 ± 21.35	54.28 ± 22.52	42.15 ± 19.41	0.103

**Table 3:** Quality of life compared with BSI grade BSI: bronchiectasis severity index

	BSI score			
	Low	Intermediate	High	p-Value
	Mean ± SD	Mean ± SD	Mean ± SD	
Physical	57.91 ± 19.68	51.78 ± 34.34	31.23 ± 22.97	0.008
Role	61.67 ± 22.75	59.22 ± 21.43	41.74 ± 19.61	0.017
Vitality	57.19 ± 19.20	55.26 ± 21.42	36.72 ± 20.77	0.011
Emotional	72.22 ± 20.82	73.93 ± 22.86	52.89 ± 19.88	0.010
Social	68.75 ± 21.81	53.52 ± 20.50	54.71 ± 20.07	0.216
Treatment burden	56.30 ± 31.82	73.66 ± 31.98	62.51 ± 17.07	0.352
Respiratory symptoms	79.50 ± 15.28	57.19 ± 25.43	49.96 ± 20.79	<0.001
Health perceptions	54.95 ± 24.62	61.67 ± 14.06	42.08 ± 19.55	0.035

In the physical scale of QOL-B, multivariate regression analysis for predictors of low score showed associations with 6MWD and BMI. There were no associations with MMRC, CRP, FEV1, colonizations, FACED scores, and BSI scores (Table 4). 

**Table 4:** Factors affecting QOL physical MMRC: Modified Medical Research Council, CRP: C-reactive protein, BMI: body mass index, BSI: bronchiectasis severity index, CI: confidence interval; FACED: FEV1, age, colonization, extension, dyspnea; QOL: quality of life

	Simple linear regression			Multiple linear regression		
	β coefficient	95% CI	p-Value	β coefficient	95% CI	p-Value
MMRC	-14.17	-20.16 to -8.17	<0.001	1.75	-8.21 to 11.71	0.723
6-minute walk test	0.17	0.11 to 0.22	<0.001	0.18	0.08 to 0.29	0.001
CRP	-1.57	-3.91 to 0.71	0.182	0.85	-1.39 to 3.09	0.447
FEV1	0.63	0.28 to 0.97	0.001	0.17	-0.31 to 0.66	0.473
BMI	1.56	-1.19 to 4.31	0.258	-2.91	-5.67 to -0.15	0.039
Colonization	-13.84	-29.76 to 2.08	0.087	-5.51	-21.74 to 10.73	0.496
FACED	-6.42	-9.73 to -3.10	<0.001	-1.92	-8.23 to 4.39	0.541
BSI	-2.83	-4.13 to -1.53	<0.001	-0.24	-2.73 to 2.26	0.848

In the role scale of QOL-B, multivariate regression analysis for predictors of low score showed association with 6MWD. There were no associations with MMRC, CRP, FEV1, BMI, colonizations, FACED scores, and BSI scores (Table 5).

**Table 5:** Factor affecting QOL role MMRC: Modified Medical Research Council, CRP: C-reactive protein, BMI: body mass index, BSI: bronchiectasis severity index, CI: confidence interval; FACED: FEV1, age, colonization, extension, dyspnea; QOL: quality of life

	Simple linear regression			Multiple linear regression		
	β coefficient	95% CI	p-Value	β coefficient	95% CI	p-Value
MMRC	-7.70	-13.48 to -1.92	0.010	7.09	-2.69 to 16.88	0.150
6-minute walk test	0.11	0.06 to 0.17	<0.001	0.12	0.01 to 0.23	0.028
CRP	-0.89	-2.88 to 1.09	0.369	0.97	-1.24 to 3.17	0.379
FEV1	0.45	0.15 to 0.75	0.005	0.17	-0.31 to 0.65	0.479
BMI	1.85	-0.46 to 4.12	0.108	-1.24	-3.95 to 1.48	0.361
Colonization	-4.77	-0.18 to 9.02	0.489	4.48	-11.46 to 20.42	0.572
FACED	-4.66	-7.58 to -1.75	0.002	-0.80	-7.01 to 5.40	0.794
BSI	-2.16	-3.29 to -1.02	<0.001	-1.74	-4.20 to 0.71	0.157

In the vitality scale of QOL-B, multivariate regression analysis for predictors of low score showed associations with 6MWD. There were no associations with MMRC, CRP, FEV1, BMI, colonizations, FACED scores, and BSI scores (Table 6). 

**Table 6:** Factor affecting QOL vitality MMRC: Modified Medical Research Council, CRP: C-reactive protein, BMI: body mass index, BSI: bronchiectasis severity index, CI: confidence interval; FACED: FEV1, age, colonization, extension, dyspnea; QOL: quality of life

	Simple linear regression			Multiple linear regression		
	β coefficient	95% CI	p-Value	β coefficient	95% CI	p-Value
MMRC	-9.97	-15.34 to -4.59	0.097	4.05	-4.88 to 12.99	0.364
6-minute walk test	0.14	0.09 to 0.19	<0.001	0.17	0.07 to 0.26	0.001
CRP	-1.51	-3.44 to 0.43	0.125	0.86	-1.15 to 2.87	0.391
FEV1	0.43	0.13 to 0.73	0.006	0.02	-0.42 to 0.46	0.923
BMI	2.45	0.25 to 4.65	0.30	-0.87	-3.34 to 1.61	0.483
Colonization	-11.21	-24.54 to 2.11	0.097	-6.09	-20.66 to 8.47	0.401
FACED	-4.99	-7.82 to -2.15	0.001	-2.19	-7.86 to 3.47	0.437
BSI	-2.23	-3.33 to -1.11	<0.001	0.05	-2.19 to 2.29	0.967

In the emotional scale of QOL-B, multivariate regression analysis for predictors of low score showed association with 6MWD. There were no associations with MMRC, CRP, FEV1, BMI, colonizations, FACED scores, and BSI scores (Table 7).

**Table 7:** Factor affecting QOL emotional MMRC: Modified Medical Research Council, CRP: C-reactive protein, BMI: body mass index, BSI: bronchiectasis severity index, CI: confidence interval; FACED: FEV1, age, colonization, extension, dyspnea; QOL: quality of life

	Simple linear regression			Multiple linear regression		
	β coefficient	95% CI	p-Value	β coefficient	95% CI	p-Value
MMRC	-9.49	-15.06 to -3.92	0.001	-1.09	-10.80 to 8.62	0.821
6-minute walk test	0.13	0.07 to 0.18	<0.001	0.96	-0.01 to 0.20	0.072
CRP	-2.56	-4.42 to -0.70	0.008	-1.12	-3.30 to 1.07	0.306
FEV1	0.28	-0.04 to 0.61	0.084	-0.31	-0.78 to 0.17	0.200
BMI	2.92	0.73 to 5.09	0.010	-0.15	-2.84 to 2.54	0.911
Colonization	-10.19	-23.79 to 3.40	0.138	2.96	-12.86 to 18.79	0.706
FACED	-4.45	-7.42 to -1.48	0.004	-250	-8.66 to 3.66	0.415
BSI	-2.18	-3.32 to -1.03	<0.001	-0.47	-2.90 to 1.97	0.700

In the social scale of QOL-B, multivariate regression analysis for predictors of low score showed that there were no associations with 6MWD, MMRC, CRP, FEV1, BMI, colonizations, FACED scores, or BSI scores (Table 8).

**Table 8:** Factor affecting QOL social MMRC: Modified Medical Research Council, CRP: C-reactive protein, BMI: body mass index, BSI: bronchiectasis severity index, CI: confidence interval; FACED: FEV1, age, colonization, extension, dyspnea; QOL: quality of life

	Simple linear regression			Multiple linear regression		
	β coefficient	95% CI	p-Value	β coefficient	95% CI	p-Value
MMRC	-7.32	-13.65 to -0.99	0.024	2.57	-9.37 to 14.50	0.665
6-minute walk test	0.09	0.02 to 0.15	0.011	0.11	-0.02 to 0.24	0.103
CRP	-0.77	-2.91 to 1.37	0.472	0.45	-2.23 -3.13	0.735
FEV1	0.36	0.02 to 0.69	0.039	0.21	-0.38 to 0.79	0.474
BMI	0.23	-2.29 to 2.74	0.857	-2.44	-5.75 to 0.86	0.142
Colonization	-7.67	-22.39 to 7.04	0.299	-2.56	-22.01 to 16.88	0.791
FACED	-3.20	-6.56 to 0.15	0.061	0.32	-7.24 to 7.88	0.932
BSI	-1.57	-2.90 to -0.24	0.022	-0.73	-3.72 to 2.26	0.623

In the treatment burden scale of QOL-B, multivariate regression analysis for predictors of low score showed associations with 6MWD and FEV1. There were no associations with MMRC, CRP, BMI, colonizations, FACED scores, and BSI scores (Table 9). 

**Table 9:** Factor affecting QOL treatment burden MMRC: Modified Medical Research Council, CRP: C-reactive protein, BMI: body mass index, BSI: bronchiectasis severity index, CI: confidence interval; FACED: FEV1, age, colonization, extension, dyspnea; QOL: quality of life

	Simple linear regression			Multiple linear regression		
	β coefficient	95% CI	p-Value	β coefficient	95% CI	p-value
MMRC	3.13	-4.46 to 10.73	0.408	7.98	-5.81 to 21.77	0.246
6-minute walk test	0.02	-0.05 to 0.09	0.548	0.16	0.03 to 0.29	0.017
CRP	-0.15	-3.11 to 2.81	0.919	-0.69	-4.16 to 2.78	0.687
FEV1	-0.43	-0.79 to -0.07	0.021	-0.74	-1.38 to -0.11	0.023
BMI	-0.87	-3.64 to 1.90	0.528	-2.55	-5.86 to 0.75	0.125
Colonization	-1.79	-19.16 to 15.57	0.835	-4.24	-0.27 to 18.65	0.707
FACED	2.08	-1.73 to 5.90	0.275	-2.92	-10.73 to 4.89	0.451
BSI	0.29	-1.27 to 1.84	0.708	0.16	-2.94 to 3.27	0.915

In the respiratory symptoms scale of QOL-B, multivariate regression analysis for predictors of low score showed association with 6MWD. There were no associations with MMRC, CRP, FEV1, BMI, colonizations, FACED scores, and BSI scores (Table 10).

**Table 10:** Factor affecting QOL respiratory symptoms MMRC: Modified Medical Research Council, CRP: C-reactive protein, BMI: body mass index, BSI: bronchiectasis severity index, CI: confidence interval; FACED: FEV1, age, colonization, extension, dyspnea; QOL: quality of life

	Simple linear regression			Multiple linear regression		
	β coefficient	95% CI	p-Value	β coefficient	95% CI	p-Value
MMRC	-14.44	-19.53 to -9.35	<0.001	-1.72	-10.19 to 6.76	0.684
6-minute walk test	0.17	0.12 to 0.22	<0.001	0.11	0.02 to 0.20	0.018
CRP	-2.09	-4.17 to -0.01	0.049	0.37	-1.54 to 2.27	0.699
FEV1	0.62	0.31 to 0.92	<0.001	0.15	-0.27 to 0.56	0.481
BMI	3.12	0.77 to 5.48	0.010	-0.48	-2.83 to 1.87	0.682
Colonization	-10.88	-25.54 to 3.78	0.142	2.98	-10.84 to 16.79	0.664
FACED	-6.39	-9.31 to -3.47	<0.001	0.91	-4.48 to 6.29	0.733
BSI	-3.08	-4.14 to -2.02	<0.001	-1.63	-3.76 to 0.50	0.129

In the health perceptions scale of QOL-B, multivariate regression analysis for predictors of low score showed association with 6MWD. There were no associations with MMRC, CRP, FEV1, BMI, colonizations, FACED scores, and BSI scores (Table 11).

**Table 11:** Factor affecting QOL health perceptions MMRC: Modified Medical Research Council, CRP: C-reactive protein, BMI: body mass index, BSI: bronchiectasis severity index, CI: confidence interval; FACED: FEV1, age, colonization, extension, dyspnea; QOL: quality of life

	Simple linear regression			Multiple linear regression		
	β coefficient	95% CI	p-Value	β coefficient	95% CI	p-Value
MMRC	-6.44	-12.03 to -0.85	0.025	4.79	-4.75 to 14.34	0.315
6-minute walk test	0.11	0.06 to 0.16	<0.001	0.16	0.06 to 0.27	0.003
CRP	-1.55	-3.39 to 0.29	0.097	0.04	-2.10 to 2.19	0.967
FEV1	0.18	-0.13 to 0.49	0.252	-0.20	-0.67 to 0.27	0.387
BMI	1.71	-0.45 to 3.87	0.118	-1.29	-3.93 to 1.36	0.330
Colonization	-9.46	-22.29 to 3.36	0.144	-4.64	-20.19 to 10.91	0.549
FACED	-2.92	-5.87 to 0.04	0.053	-2.08	-8.13 to 3.97	0.490
BSI	-1.58	-2.73 to -0.42	0.009	0.05	-2.34 to 2.45	0.964

## Discussion

This study looked at the QOL-B scores and their various domains in patients with NCFB from a tertiary care center in South India. From an extensive review of the literature, no other similar studies could be identified and this is potentially the first such study from this geographical region. Our study is also unique in the fact that it included patients with very well-defined bronchiectasis who prospectively underwent multiple systems of scoring for the severity of NCFB including the BSI and FACED scores. They then prospectively were assessed for QOL-B scores, as well as outcome parameters including MMRC grading, BMI, 6MWD, CRP, and FEV1. This study demonstrated a significant impact on the QOL of NCFB patients based on the QOL-B scoring system. Almost all domains of QOL-B were found to be adversely impacted as measured by one or more of the outcome parameters but the FACED score, BSI score, 6MWD, and FEV1 showed associations across most scales while the other outcome parameters showed varying associations. 

The 6MWD was found to correspond inversely with almost all domains of the QOL-B except for the treatment burden domain. 6MWD is a simple exercise test to evaluate the general functional responses of the pulmonary, cardiovascular, as well as muscular systems to assess daily physical activities and QOL of patients with NCFB. A study by Hsieh et al. reported that poorer 6MWD test results correlating with increased mortality in NCFB indicating that 6MWD may be an important predictor of QOL as well [6]. However, Jacques et al. reported otherwise in their study looking specifically at the role of 6MWD as an indicator of QOL in NCFB, stating that it was not a measure of QOL [7]. Given the highly ambiguous findings in previous literature, we believe that this study serves to add support to the 6MWD in the assessment of QOL in NCFB. 

FEV1 predicted was also found to correspond inversely with almost all domains of the QOL-B except for the Health Perceptions domain. A large meta-analysis by Habib et al. reported that significant associations with FEV1% predicted and QOL in NCFB [8]. These findings were corroborated in this study. The BSI score also showed a homogenous association across domains, with higher BSI grades corresponding to lower QOL domain scores. This was in keeping with previously reported data by McDonnell et al. [9]. The FACED score also showed similar homogenous association across QOL-B domains, with higher FACED grades corresponding to lower QOL domain scores, however, McDonnell et al. described various limitations in the use of FACED score in the assessment of QOL in NCFB [9]. The other parameters of QOL-B showed varying associations with the outcome parameters studied, with the heterogeneity of findings indicating limited clinical utility. 

There were some limitations in this study. First, the number of patients enrolled in this study was limited, and all the patients were recruited from a single-center, which can limit the generalizability of the results of this study. Secondly, transversal variables related to NCFB, such as pulmonary hypertension or cardiovascular disorders and its impact on quality of life, were not included in the study due to the limited number of patients. Thirdly, evolutionary variables, including the number of exacerbations or hospitalizations due to NCFB or otherwise which could impact certain domains of QOL-B, were not included in the analysis. Finally, the impact of non-tuberculosis mycobacterial infection or colonization in these patients could not be studied. Hence, larger, multi-center studies may be needed with larger numbers of patients to corroborate our findings.

## Conclusions

The QOL is significantly reduced in NCFB and it may be quantified using the QOL-B questionnaire. The impact on QOL in NCFB may be assessed using validated tools such as the FACED and BSI scoring systems, as well as other well-established outcome parameters like 6MWD and FEV1 predicted. The role of outcome parameters such as BMI, CRP, MMRC, and colonization may be limited individually but may play a significant role when used in combinations.
